# Incidence of Symptomatic Vertebral Fractures Among Newly Diagnosed Autoimmune Diseases Initiating Glucocorticoid Therapy

**DOI:** 10.1097/MD.0000000000000875

**Published:** 2015-07-13

**Authors:** Kiyoshi Migita, Nozomi Iwanaga, Shunsuke Imadachi, Yuka Jiuchi, Yasumori Izumi, Yoshika Tsuji, Chieko Kawahara, Atsushi Kawakami, Hiroshi Furukawa, Shigeto Tohma

**Affiliations:** From the Department of General Internal Medicine and Rheumatology, Clinical Research Center, NHO Nagasaki Medical Center, Omura, Nagasaki (KM, NI, SI, YJ, Yi, YT, CK); Japanese National Hospital Organization (NHO)-EBM Study Group for Adverse Effects of Corticosteroid Therapy (J-NHOSAC), Hikarigaoka, Meguro, Tokyo (KM); Department of Rheumatology, Unit of Translational Medicine, Graduate School of Biomedical Sciences, Nagasaki University, Nagasaki (AK); and Clinical Research Center for Allergy and Rheumatology, National Hospital Organization, Sagamihara, Japan (HF, ST).

## Abstract

Few data are available regarding vertebral fracture risk in patients treated with corticosteroids including patients with interstitial lung disease (ILD). The aim of the present study was to identify risk factors for symptomatic vertebral fracture analyzed in patients with newly diagnosed autoimmune diseases.

This was an observational cohort study conducted in the National Hospital Organization-EBM study group from 2006 to 2008. The study subjects were autoimmune disease patients who were newly treated with glucocorticoids (GCs). The primary endpoint was the first occurrence of vertebral fracture diagnosed by x-rays. Cox proportional-hazards regression was used to determine independent risk factors for vertebral fracture with covariates including sex, age, comorbidity, laboratory data, use of immunosuppressants, and dose of GCs. Survival was analyzed according to the Kaplan–Meier method and assessed by the log-rank test.

Among 604 patients of mean age 59.5 years and mean GC dose 50.4 mg/d (first 1 months), 19 patient (3.1%) had at least 1 symptomatic vertebral fracture during 1.9 years of follow-up period. Cox regression model demonstrated that the relative risk for symptomatic vertebral fracture was independently higher in patient with ILD (hazard ratio [HR] = 2.86, 95% confidence interval [CI] = 1.10–7.42, *P* = 0.031) and in every 10-year increment of the age of disease onset (HR = 1.57, 95% CI = 1.09–2.26, *P* = 0.015). Kaplan–Meier analyses demonstrated that the incidence of vertebral fractures in patients with ILD was significantly higher in comparison with those without ILD.

Our results indicate a higher risk of vertebral facture in patients with ILD and elderly patients during the initial GC treatment against autoimmune diseases. There is a need for further, even longer-term, prospective studies subjected patients with autoimmune disease, including ILD, under GC treatment.

## INTRODUCTION

Glucocorticoids (GCs) have been widely used in patients with autoimmune lung disease, as well as in patients with interstitial lung disease (ILD).^1^ Fractures, as a result of osteoporosis, are serious complications in patients receiving GCs.^2^ Several large-scale cross-sectional and meta-analysis have demonstrated that GCs treatment induced osteoporosis and increased the risks for vertebral fractures.^3^ Additionally, many randomized clinical trial have demonstrated that initiation of bisphosphonates prevents bone loss and fracture.^4^ However, it is unlikely that a definite clinical trial regarding fracture will be performed for many reasons, including relatively low frequency of fractures as an outcome and difficulty recruiting enough eligible patients for a trial to be sufficiently powered.^4^

Autoimmune diseases appear to be conditions that are particularly associated with decreased bone mineral density (BMD) and increased fractures, which may be partly iatrogenic and preventable.^5^ There are several potential iatrogenic and lifestyle contributions to osteoporosis. Iatrogenic factors include adverse bone-related biochemical effects of corticosteroids and immunosuppressant, which have been demonstrated in animal studies, induce bone resorption, and cause osteoporosis.^6^ Relevant lifestyle factors may lead to adverse bone effects including reduced levels of physical activity and bone-related biochemical effects associated with cigarette smoking and alcohol consumption.^7^ Also in subjects with severe autoimmune diseases, periods of greatly reduced activity during hospitalization may lead to accelerated bone loss.^8^ Additionally, patients treated with GC may progress bone loss, with very different types of underlying various autoimmune diseases, relating to their disease processes and various organ involvements.^9^ Therefore, the cohort against various autoimmune diseases patients treated with GC study should be conducted to determine the actual incidence of fractures and the preventive effects of various antiosteoporosis agents under real-world setting. In the present study, we investigated the risk factors of symptomatic vertebral fracture, analyzing the database of cohort study conducted on patients with recently diagnosed autoimmune disease in the National Hospital Organization (NHO) hospitals between 2006 and 2008.^10^

## METHODS

### Patients and Study Design

Patients were eligible for the study if they were initially treated with GCs for a recently diagnosed (within 4 weeks prior to study entry) autoimmune disease using established criteria.^10^ The cohort start date was defined as the time of initiation of the first GC prescription. The autoimmune diseases registered in this study were as follows: rheumatic diseases—systemic lupus erythematosus, mixed connective-tissue disease, polymyositis, dermatomyositis, vasculitis, Behçet disease, systemic scleroderma, adult-onset Still disease, Sjögren syndrome, rheumatoid arthritis, autoimmune bullous diseases, and anaphylactoid purpura; neurological diseases—multiple sclerosis, myasthenia gravis, and chronic inflammatory demyelinating polyneuropathy; gastrohepatobiliary diseases—ulcerative colitis, autoimmune hepatitis, autoimmune pancreatitis, and primary biliary cirrhosis. A diagnosis of ILD was established according to the criteria of the ATS, including consistent clinical features and pulmonary function tests, radiographic evidence of interstitial disease, and/or lung histopathology consistent with this diagnosis.^11^ All patients were evaluated for ILD using high-resolution computed tomography (HRCT) scans of their lungs. Diagnosis of ILD was determined by a panel of ILD expert clinicians and chest radiologists based on serology, clinical signs, and HRCT analysis. connective tissue diseases-ILD was diagnosed if ILD was found in the presence of rheumatic disease.^12^ Primary glomerular diseases include rapidly progressive glomerulonephritis, chronic glomerulonephritis, and nephrotic syndrome.

A total of 604 patients with newly diagnosed autoimmune disease were enrolled between April 1, 2006, and March 31, 2008, and regularly followed concerning the occurrence of GC-related adverse effects (AEs). The observation period ended on March 31, 2009.

### Ethics Approval

Before implementation of this study, institutional review board and ethics committee approvals of the protocol and the consent to participate and publish the study were obtained from each of the participating patients. The study was approved by the ethical committees of the NHO central internal review board (No. 0512014, 2006). Written informed consent was obtained from each individual.

### Data Collection

Data from all participating physicians were entered into the Japanese National Hospital Organization, Study for Adverse Effects of Corticosteroid Therapy (J-NHOSAC) database at the data center of the International Medical Center of Japan in Tokyo, Japan, via the HOSPnet Internet system.

### Data on Study Entry

The past comorbid condition of each patient was reviewed by each of the principle physicians. These conditions included renal, neurological, endocrine, cardiovascular, and pulmonary diseases as well as cancer and stroke. In addition, the incidences of specific conditions, including preexisting pulmonary tuberculosis (TB), hepatitis viral infection (hepatitis B virus, hepatitis C virus), diabetes, hyperlipidemia, arrhythmia, and performance status (Karnofsky score), were assessed. The physicians also provided information on smoking or drinking habits and history of TB.

### Outcome Variables

At the start of the study, standardized lists were used to document AEs, which were classified using the System Organ Class of the Medical Dictionary for Regulatory Activities (MedDRA; version 11.1). Each patient received structured medical interview, including symptoms suggesting vertebral fractures such as back pain, or lumbago and performance status assessed by Karnofsky score. All physicians documented episodes of GC-related AEs requiring medical care and death certificates and the causes of deaths that occurred during the follow-up periods. Patients were followed up every 3 months by the chief physician for each of the NHO hospitals, who collected clinical findings (disease activity, severity, performance status, blood pressure, and body weight) and laboratory data (complete blood cell count, biochemistry, and urinalysis). The telephone interview concerning the health assessment and the presence of GCs-related AEs was conducted against few patients who were moved or transferred to another hospital at the end of the cohort. However, overall outcome was not available from 2 patients (2/133, 1.5%) at the end of the study.

### Symptomatic Vertebral Fracture

The primary endpoint was the first occurrence of a diagnosed symptomatic vertebral fracture. When symptoms suggestive of vertebral fracture occurred, patients underwent radiography according to the standardized protocol defined in this study. Symptomatic vertebral fracture was defined as vertebral deformity with a clinical symptom that was confirmed by thoracolumbar x-rays in patients with a backache. Lateral thoracolumbar x-rays of the spine were graded independently by a radiologist. A symptomatic vertebral fracture was defined as vertebral deformity that was confirmed by lateral thoracolumbar radiography in patients with sudden severe backache. Vertebral deformity was assessed semiquantitatively by method similar to that described by Genant et al,^13^ and deformity was defined to be present if there was >20% reduction in anterior, middle, or posterior vertebral height.

### Follow-Up Data

Patients were followed up every 3 months by the chief physician for each of the NHO hospitals, who collected clinical findings (disease activity, severity, performance status, blood pressure, and body weight) and laboratory data (complete blood cell count, biochemistry, and urinalysis). The telephone interview concerning the health assessment and the presence of GCs-related AEs was conducted against few patients who were moved or transferred to another hospital at the end of the cohort. However, overall outcome was not available from 14 patients (2.3%) at the end of the study. In statistical analysis, we excluded these participants without final outcome data.

### Medications

Details of GCs, immunosuppressants, and biologics were recorded at each visit, including the route of administration and dose. We categorized GC exposure according to the mean daily dose throughout the follow-up period for each patient. We calculated “dose equivalents” of prednisolone as follows: 1 mg of prednisolone = 5 mg of cortisone = 4 mg of hydrocortisone = 1 mg of prednisone = 0.8 mg of triamcinolone = 0.8 mg of methylprednisolone = 0.15 mg of dexamethasone = 0.15 mg of betamethasone.^14^

### Statistical Analysis

Qualitative variables were compared using the χ^2^ test (or Fisher exact test when appropriate), and quantitative variables were compared using the Mann–Whitney *U* test. The incidence rate of fracture was calculated as the number of fractures per 1000 patient-years (PY). We identified the risk factors of vertebral fracture by univariate and multivariate Cox-proportional hazard models analysis. The variables included in the analysis were age, sex, types of primary autoimmune diseases, comorbidities (diabetes, renal diseases, cardiovascular diseases, and ILDs), medications (average dose of GC, the use of immunosuppressive agents), and performance status (Karnofsky score). Factors included in the Cox-proportional multivariate hazard model were those associated with the status (case or control) of univariate analysis with a significance level of *P* < 0.20. Both univariate and multivariate analyses were performed, using the occurrence of at least 1 vertebral fractures during the follow-up period as the outcome. Results are expressed as odds ratios with 95% confidence intervals (95% CIs). Survival, related to follow-up time, was analyzed using the Kaplan–Meier method and compared using the log-rank test. Two-sided *P* values <0.05 were considered statistically significant. The analysis was conducted using SAS (version 9.1; SAS Institute, Cary, NC).

## RESULTS

### Clinical Characteristics of the Study Population

The baseline characteristics are shown in Table 1. A total of 604 patients with newly diagnosed autoimmune diseases initially treated with GCs from 51 hospitals were enrolled in the J-NHOSAC registry between April 2006 and March 2008. The analysis was performed on all patients with a mean follow-up period of 1.9 ± 0.64 years, for a total of 1105.8 PY. There were 358 (59.3%) females and 246 (40.7%) males, with a mean age of 59.5 ± 16.8 (range 17–94) years. All patients received GCs at entry and the mean GCs dose for the first month was 50.4 ± 63.1 mg/d. Concomitant immunosuppressive therapies are summarized in Table 1. Enrolled patients were categorized into the 5 groups according to the primary autoimmune diseases (Table 2). There were some variations in the age of the onset of primary diseases, sex, initial mean dose of GC, performance status evaluated by Karnofsky score, and the presence of comorbidity. In patients with idiopathic interstitial pneumonias (IIPs), the elderly onset of primary disease, higher mean initial dose of GC, higher comorbidities, and lower performance status were observed compared to other groups of patients.

**TABLE 1:**
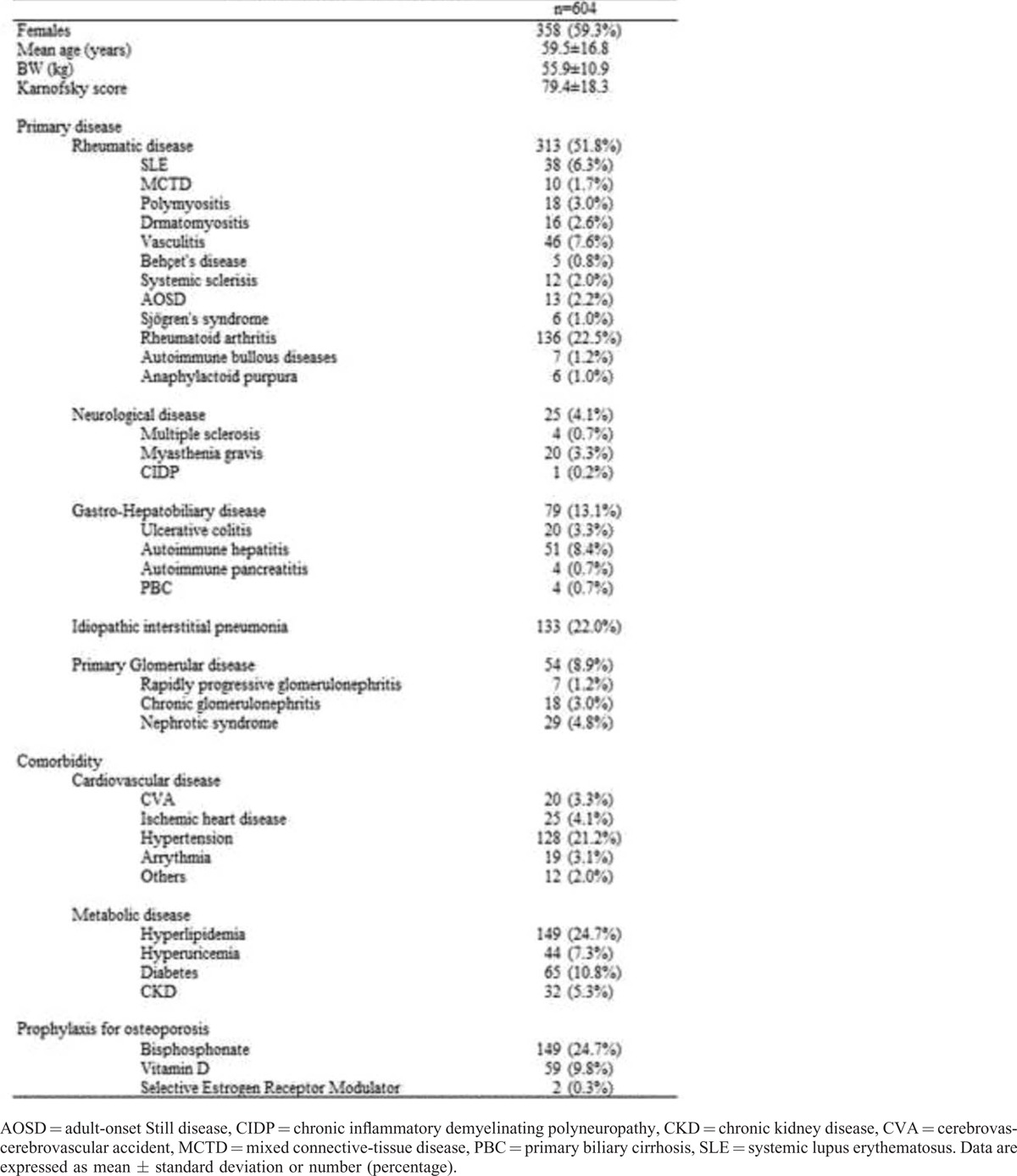
Baseline characteristics of the 604 patients with autoimmune diseases treated with glucocorticoid

**TABLE 2:**
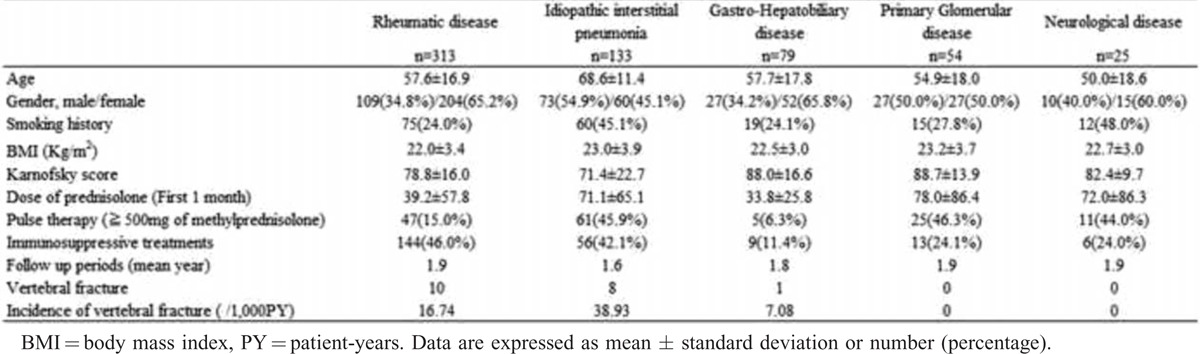
Baseline clinical and demographic of primary disease group

### Incidence of Vertebral Fracture

**FIGURE 1:**
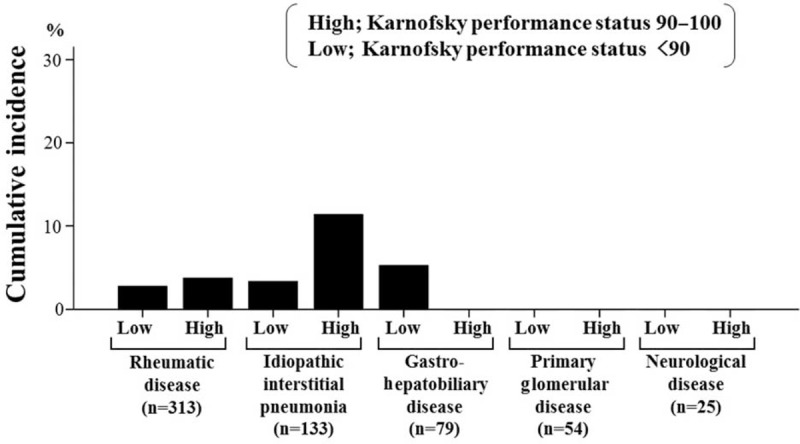
Incidences of symptomatic vertebral fractures according to the performance status. Incidences of symptomatic vertebral fractures in each groups of patients stratified according to the performance status assessed by Karnofsky score.

Despite a shorter follow-up period, these were newly observed 19 vertebral fractures. Table 2 shows a comparison of the incidence of vertebral fracture among patients’ groups. Among 604 patients, 19 patients (3.1%) had symptomatic vertebral fractures during the follow-up periods (1.8 ± 0.7 years) indicating that the overall incidences of vertebral fractures are 17.3/1000 PY. There were some variations in the frequencies of vertebral fractures in each hospital (3.1 ± 7.3%). Although there was heterogeneity in patient's background data among each group, there were more vertebral fractures in patients with IIPs. To determine whether vertebral fracture could be associated with the disability due to the severity of autoimmune diseases, we evaluated the frequencies of vertebral fracture according to the performance status in each group of primary autoimmune diseases. As shown in Figure 1, vertebral fractures were not exclusively observed in patients with lower performance status in each group of primary diseases.

### Risk Factors for Vertebral Fractures

To identify differences in risk factors that contribute to the development of vertebral fracture, we compared baseline data between patients with vertebral fracture and those without vertebral fracture (Table 3). Risk factors for vertebral fracture identified in univariate analysis were shown in Table 4. In addition to the significant variables identified by univariate analysis, we also tentatively forced the dose of corticosteroid into the Cox-proportional hazard model. In multivariate Cox regression analysis (Table 5), independent predictors of vertebral fracture were elderly age (hazard ratio [HR] = 1.57, 10-years age increment, 95% CI = 1.09–2.26) and the presence of ILD (HR = 2.86, 95% CI = 1.10–7.42). Of note, neither mean doses of initial GCs (first 1 month) nor the use of bisphosphonates were identified as independent predictors for vertebral fracture. All patients were stratified according to the use of bisphosphonates and analyzed by multivariate analysis. Similarly, elderly age and the presence of ILD were isolated as independent predictors for vertebral fracture in patients without the use of bisphosphonates (Table 6).

**TABLE 3:**
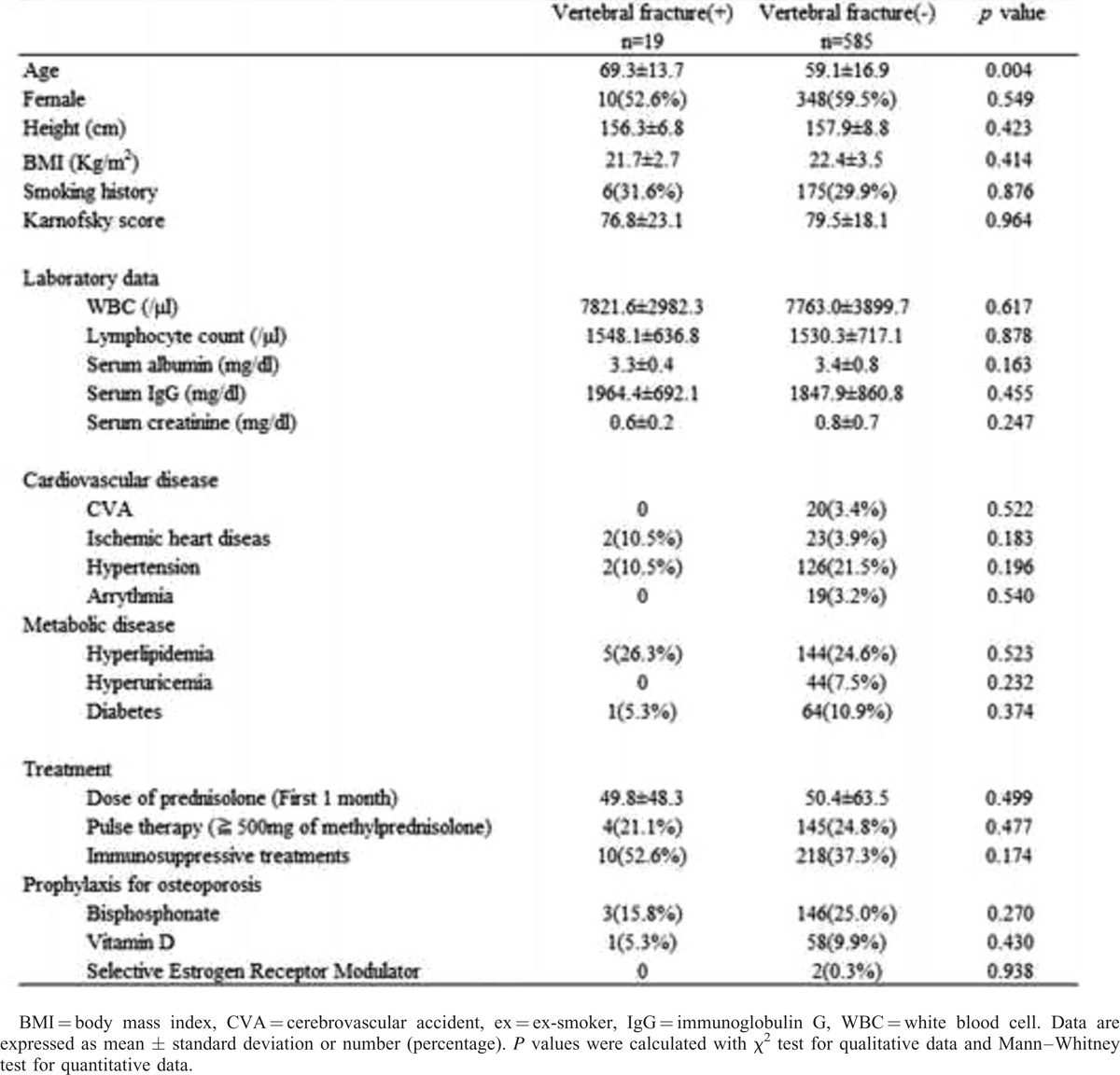
Baseline clinical and demographic vertebral features of all patients

**TABLE 4:**
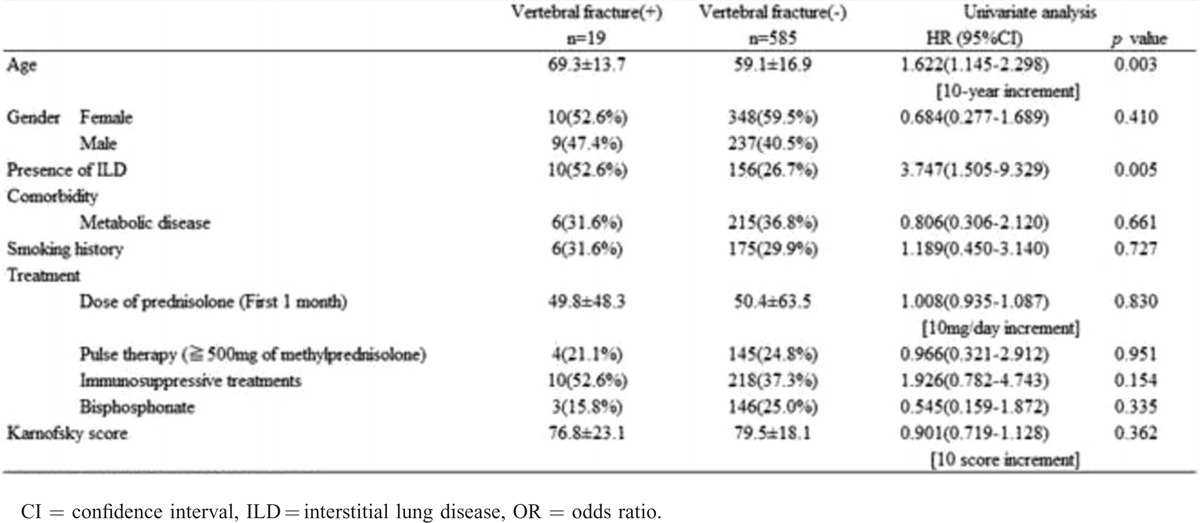
Predictors of vertebral features in the COX-hazard model (Univariate analysis)

**TABLE 5:**
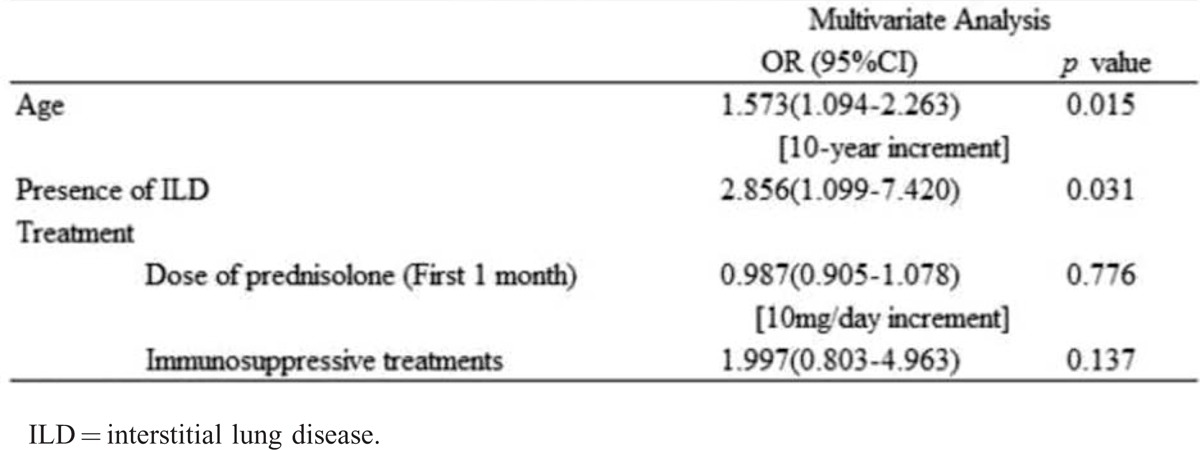
Predictors of vertebral features in the COX-hazard model (Multivariate Analysis)

**TABLE 6:**
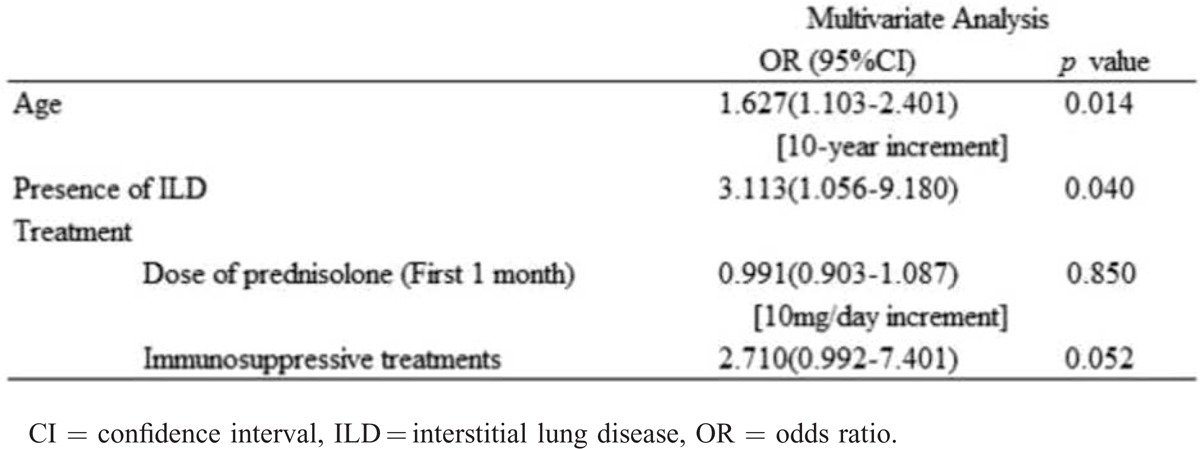
Predictors of vertebral features in the COX-hazard model (patients without the use of bisphosphonates)

**FIGURE 2:**
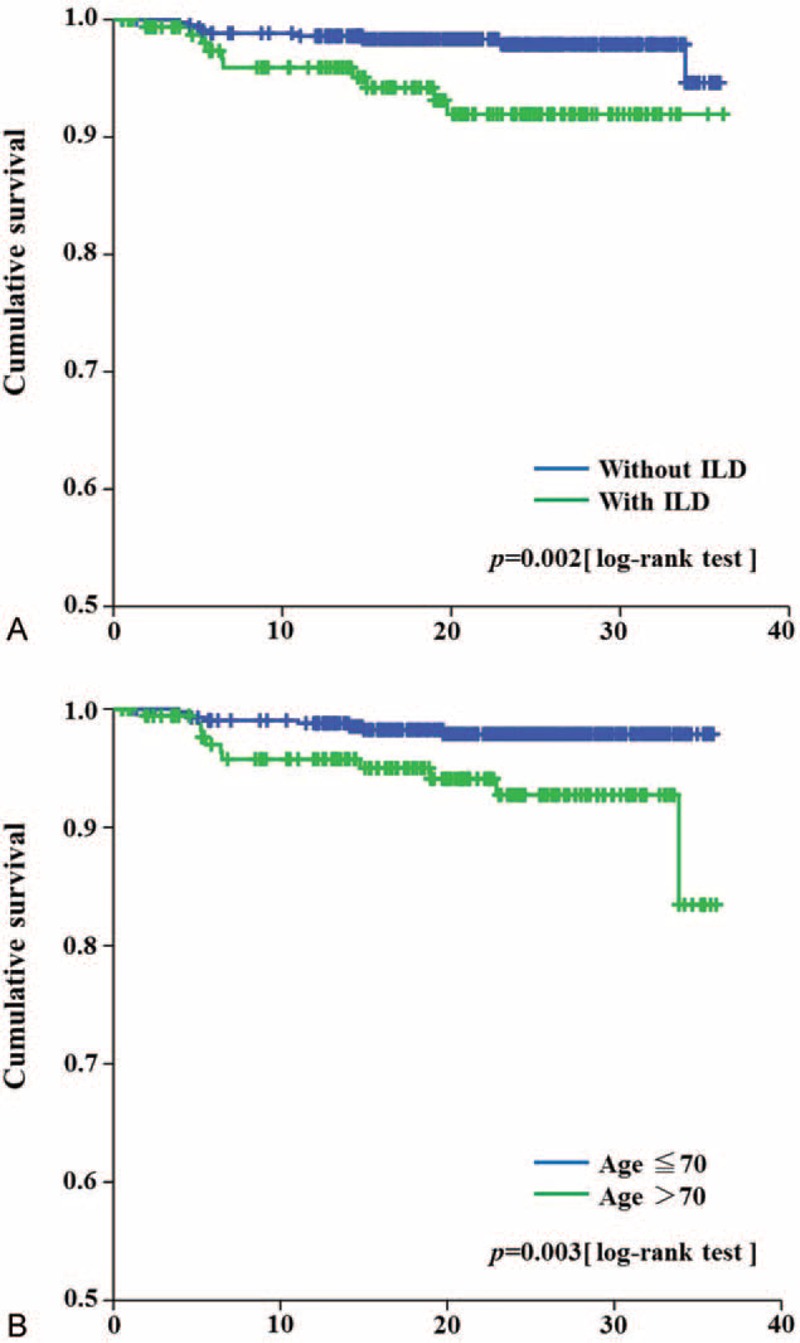
(A) Kaplan–Meier curves of fracture-free survival of patients with or without ILD. Curves are stratified by the presence or absence of ILD. Statistically significant differences were observed between patients with or without ILD (*P* < 0.0001, log-rank test). (B) Kaplan–Meier curves of fracture-free survival of patients stratified by aging. Curves are stratified by elderly age (70 years old vs ≤70 years old). Statistically significant differences were observed between these 2 groups (*P* < 0.0001, log-rank test). ILD = interstitial lung disease.

Patients with vertebral fracture were found to be significantly associated with elderly age and the presence of ILD. Therefore, Kaplan–Meier survival curves were plotted for the occurrence of the first vertebral fracture stratified by the presence of ILD and aging. Kaplan–Meier curves for vertebral fracture-free survival in patients with ILD versus patients without ILD are shown in Figure 2A. Compared to patients without ILD, the incidence of vertebral fractures was significantly higher in patients with ILD (*P* = 0.002, log-rank test, Figure 2A). Similarly, elderly age (>70 years) significantly affected the vertebral-free survival of the enrolled patients (*P* = 0.003, log-rank test, Figure 2B).

## DISCUSSION

Osteoporosis is a well-known, dose-dependent adverse effect of GC therapy.^15^ Patients with autoimmune diseases treated with GCs are at risk for osteoporosis or bone fracture,^16^ but the relative contribution of their primary disease, decreased exercise, and other factors toward vertebral fractures has not been established. Understanding the relative contribution of demographic and clinical variables to vertebral fractures in patients with autoimmune diseases may augment the prediction of fracture risk in these patients. This multicenter cohort study looked at the relationship between primary autoimmune diseases and vertebral fracture risk during initial GC treatments. We evaluated the contribution of clinical and demographic variables to vertebral fracture in patients with autoimmune diseases by using NHO cohort database.^10^ These subjects have received initial GC treatment against newly diagnosed autoimmune diseases. Our results revealed that elderly age and ILD itself are major risk factors for vertebral fracture after GC treatment. We found a clear relation between ILD and GC-induced vertebral fractures, which has not been previously demonstrated.

Autoimmune and inflammatory disorders can cause detrimental changes in bone metabolism that lead to osteoporosis dependent or independent to GC treatments.^17^ However, the studies investigating influences of underlying autoimmune disease on GC-induced vertebral fractures were limited. Although GCs are useful in the treatment of these conditions, they may cause a serious AE, bone fracture.^18^ Previous studies have suggested a prominent effect of chronic obstructive lung disease (COPD) on the vertebral fracture rate independent of steroid use.^19^ McEvoy et al^20^ reported that the proportion of COPD patients with vertebral fractures was 49%, 57%, and 62% for those receiving no GC, inhaled GC, or oral GC. In addition, subjects with COPD who had not been treated with GC had a relatively greater reduction in bone loss.^21^ Although an association between GC dose and vertebral fracture risk was not demonstrated in the present study, our data suggest that vertebral fractures are highly prevalent in patients with ILD, once the GC therapy is initialed. Furthermore, patients with respiratory diseases may have an increased risk for osteoporosis because of the sedentary lifestyle imposed by their pulmonary insufficiency.^22^ Therefore, patients with chronic lung diseases are at greater risk for osteoporosis and sustaining fractures than the general population, irrespective of GC use.

GCs are involved in the dynamics of bone and calcium metabolism.^23^ The inhibitory effects of GCs on bone growth are likely to be secondary to inhibition of bone formation with decreased synthesis of RNA, collagen, bone matrix components, and bone proteins.^24^ GCs have a greater effect on trabecular bone than on cortical bone and as a result cause fractures in vertebrae.^25^ Hypoxia has negative effect on bone mineralization,^26^ but whether the association is mediated through the degree of arterial oxygen saturation is unknown. Patients with COPD are at increased risk of osteoporosis and related fracture.^20^ This increased risk seems to be because of risk factors, such as smoking, advanced age, physical inactivity, and chronic inflammatory state.^27^ However, whether patients with ILDs have increased risk of osteoporosis or fracture under GC treatment had not been addressed. Patients with ILD tend to develop chronic hypoxia. The risks for vertebral fractures in chronic pulmonary diseases secondary to GC use may be greater than those in other diseases requiring GC. Increased ILD severity could be a potential confounding factor, as decreased physical activity may decrease bone loss. However, ILD severity is unlikely to be an important confounder as there was no difference in patients’ performance status evaluated by Karnofsky score between ILD patients with or without vertebral fracture.

Our results highlight the lack of attention given to the prevention of GC-induced vertebral fracture on focusing the primary diseases. Despite the well-known association of GCs and bone fractures, few studies addressed according to the primary diseases. Recent studies have demonstrated that GC-induced bone fracture can be effectively prevented by the established treatment regimens.^28^ Interestingly, patients with other underlying autoimmune diseases were at lower risk for vertebral fracture compared to those with ILD indicating that vertebral fracture could be a frequent complication in patients with ILD.

Our study has several limitations. Although the correlation between BMD and GC-induced fracture risk has been demonstrated,^2^ we have no data on BMD in the study subjects. Recently, it has been known that vertebral fracture associated with GC therapy often is asymptomatic.^29^ As the clinical observation of the condition initiated the diagnostic procedure for symptomatic vertebral fracture in the present study, the fracture incidences observed might be less than the degree of actual occurrence. The information concerning previous vertebral or fragility fractures was lacking in this study. The follow-up periods (mean 1.9 years) were relatively short in this study. Lifestyle risk factors, such as calcium/vitamin D supplements and the menopause status of individual patients, were not available in this study. The vitamin D levels have been proven to be related with vertebral fracture in various autoimmune diseases.^30^ However, these information were not available in this study. It is recommended that patients with idiopathic pulmonary fibrosis (IPF) should not be treated with corticosteroid monotherapy according to American Thoracic Society and the European Respiratory Society guidelines.^31^ However, some patients with IPF were treated with corticosteroid monotherapy. Also, the ethnic background of the patients in our study is predominantly Asian, which precludes the generalizability of the results to other ethnic populations. The number of subjected patients was relatively small and there was a deal of heterogeneity in the patient's demographic data according to the primary diseases.

In conclusion, we have shown that age and the presence of ILD are important and independent risk factors for vertebral fractures in patients newly treated with corticosteroids. Further studies should examine whether bisphosphonate or other treatment in ILD patients also produce consequent reduction in vertebral fractures noted in these patients. Given the high rate of vertebral fractures noted in ILD patients, regular screening for vertebral fractures and BMD should be considered.
